# Vancomycin is a risk factor for acute pancreatitis and increases the mortality rate in patients with severe pancreatitis

**DOI:** 10.3389/fimmu.2025.1701491

**Published:** 2026-01-12

**Authors:** Yongxiang Wang, Hongye He, Zou Heng, Zhongtao Liu, Yu Wen

**Affiliations:** 1 Department of General Surgery, Second Xiangya Hospital, Central South University, Changsha, Hunan, China; 2 Department of General Surgery, Ward 4, The Fourth Hospital of Changsha, Changsha, China

**Keywords:** intensive care unit, mediation effects, prognosis, severe pancreatitis, vancomycin

## Abstract

**Background:**

In the later stages of severe acute pancreatitis, serious infections often occur, leading to a worse prognosis. Vancomycin is beneficial for controlling infections, but it has hepatotoxic and nephrotoxic effects.

**Methods:**

Data regarding the toxic side effects of vancomycin were obtained from the FDA Adverse Event Reporting System (FAERS), and animal experiments were conducted to validate these findings.Clinical data of patients with severe pancreatitis were extracted from the MIMIC database. Subsequently, the survival and matching packages were utilized to perform propensity score matching to assess the impact of vancomycin on patient prognosis. Finally, the mediation package was employed to analyze the clinical factors that mediate the effect of vancomycin on prognosis.

**Results:**

Data from the FAERS database and animal studies suggest that vancomycin may harm pancreatic tissue. Consequently, the administration of vancomycin in patients with severe pancreatitis increases their risk of mortality. In the Intensive Care Unit (ICU), the mediation effects of potassium, total bilirubin, hemoglobin, age, and lactate on the relationship between vancomycin use and prognosis in severe pancreatitis were found to account for 7.07%, 5.58%, 6.51%, 16.68%, and 10.37% of the total effect, respectively. Furthermore, during the 28 days prior to ICU admission, the mediation effects of age, hemoglobin, red cell distribution, hematocrit, CO2 concentration, urea nitrogen, levofloxacin, creatinine, and heart failure accounted for 18.47%, 10.39%, 13.63%, 6.69%, 5.74%, 20.07%, 7.39%, -66.04%, and 6.82% of the total effect, respectively, concerning the same variables.

**Conclusion:**

Vancomycin may induce pancreatic injury and elevate the risk of mortality in patients with severe pancreatitis. Moreover, renal function, patient age, heart failure, and red blood cell-related indicators are significant mediating factors.

## Introduction

Pancreatitis is an inflammatory disease caused by various factors, including excessive alcohol intake, gallbladder diseases, hyperlipidemia, and trauma (1, 2). The clinical manifestations of acute pancreatitis can vary in severity, ranging from mild symptoms to life-threatening conditions (3). Approximately 20% of patients with acute pancreatitis may progress to severe pancreatitis (4, 5). Patients with severe pancreatitis often experience infection-related complications, with infected necrotizing pancreatitis being the most significant factor contributing to mortality (6). Protecting the intestinal mucosal barrier in patients with severe pancreatitis helps reduce bacterial translocation (7–9). While this approach can mitigate secondary infections in severe pancreatitis, it is still far from sufficient. In pancreatitis, infectious complications are adverse factors significantly affecting patients with acute pancreatiti (10–12). To control infections, reduce inflammation, and prevent the occurrence of complications, the rational use of antibiotics is crucial (13, 14).

Empirical treatment of infections in pancreatitis often involves third-generation cephalosporins or carbapenem antibiotics (15, 16). Gram-positive bacterial infections tend to gradually replace Gram-negative ones (17, 18), and this phenomenon is particularly pronounced when carbapenem antibiotics are used (19). Additionally, patients with severe pancreatitis frequently reside in the ICU, making them a high-risk population for methicillin-resistant *Staphylococcus* aureus(MRSA) (20). Previous studies suggest that patients with secondary pancreatic infections require antibiotics targeting MRSA (19, 21). On the other hand, the pathogens responsible for infected necrotizing pancreatitis are mostly of gastrointestinal origin, and these microorganisms can spread to the pancreas due to damage to the intestinal mucosal barrier (22). Therefore, the antibacterial treatment for infections in pancreatitis should target multiresistant *Enterobacteriaceae* and *Enterococcus* species (23). Similar to MRSA, *Enterococcus* has accumulated genes conferring resistance to various classes of antibiotics, including β-lactams, aminoglycosides, fluoroquinolones, and glycopeptides (24, 25).

Routine antibiotic treatment does not effectively control infections in severe pancreatitis. Several randomized controlled trials (RCTs) have shown that the routine use of prophylactic broad-spectrum antibiotics does not impact the development of systemic complications, the need for surgery, or mortality rates in patients with severe pancreatitis (13, 26). Vancomycin, a glycopeptide antibiotic, is effective against *Staphylococcus*, *Streptococcus*, and other Gram-positive bacteria. It is the drug of choice for treating infections caused by methicillin-resistant *Staphylococcus* aureus(MRSA), *Listeria*, and multidrug-resistant *Streptococcus* pneumoniae (27), and it is also an effective antibiotic for *Enterococcus* infections (28, 29). Therefore, in the process of controlling infections in severe pancreatitis, vancomycin can address the shortcomings of routine antibiotics.

Infection is a significantly high-risk factor for severe pancreatitis (30). Although vancomycin is beneficial for controlling infections in severe pancreatitis, it is known to have nephrotoxic effects, and there are currently no studies systematically exploring the impact of vancomycin on the prognosis of pancreatitis. This study primarily addresses this issue. In addition to investigating the impact of vancomycin use on the prognosis of patients with severe pancreatitis in the ICU, it further explores the clinical factors that affect the efficacy of vancomycin. We aim to provide guidance for the use of vancomycin in patients with severe pancreatitis.

## Methods

### Analysis of vancomycin side effects

**Figure 1:**
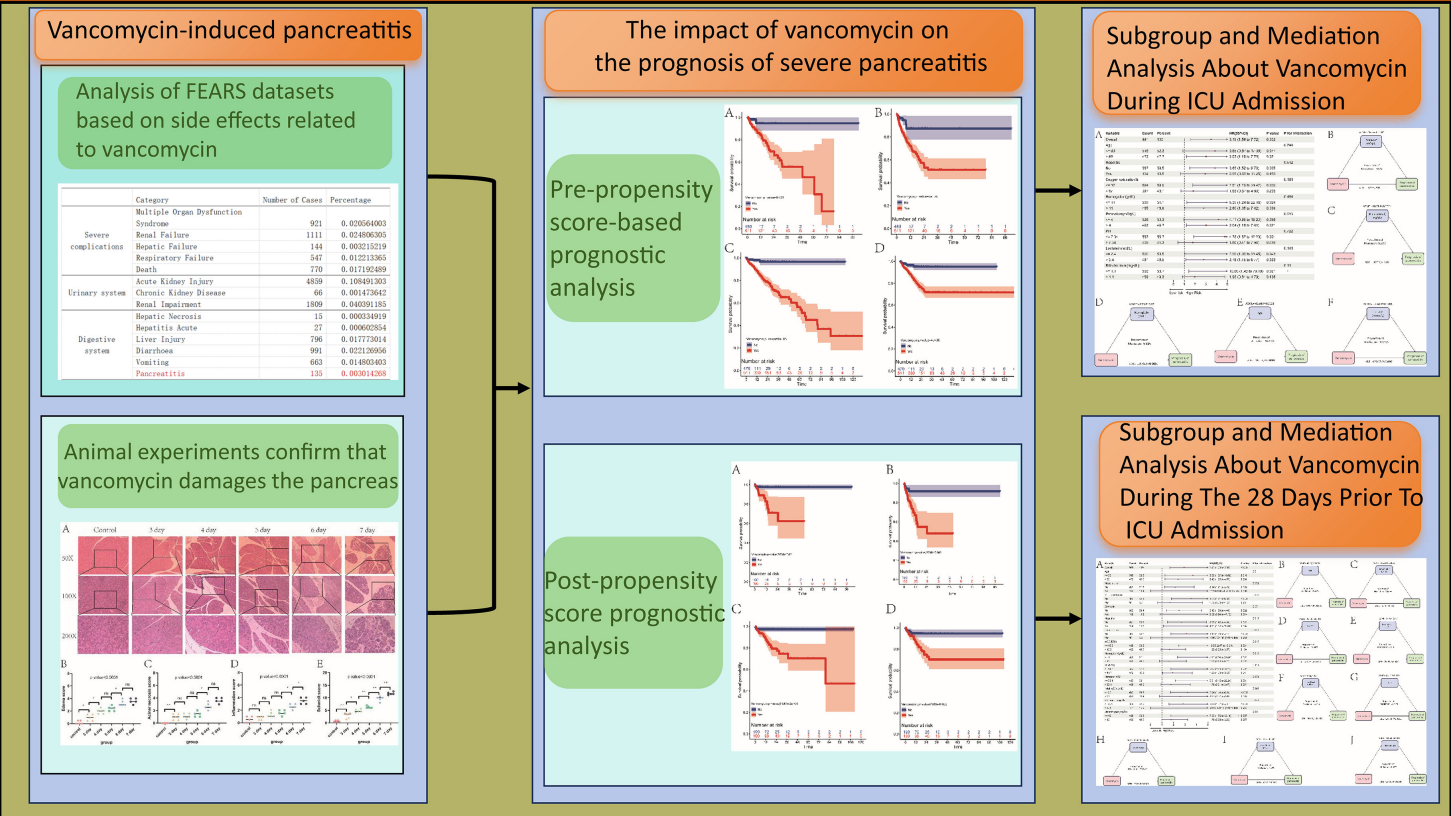
Flowchart of the study and main points.

The FDA Adverse Event Reporting System(FAERS) is a database designed to support the FDA’s post-market monitoring program for drugs and therapeutic biological products, which includes all adverse event information collected by the FDA. Both prescription and over-the-counter(OTC) medications can potentially cause side effects. In FAERS, relevant adverse reactions associated with vancomycin can be searched using “vancomycin” as a keyword.The overall experimental design and procedures of this study are illustrated in the figure (**Figure 1**).

### Animal experiment

A total of 30 male C57BL/6 mice, aged between 8 and 12 weeks, were randomly selected from five litters, with six mice from each litter. After marking each mouse with an ear tag, a random number table was generated using Excel to assign the mice to six groups: one control group and five experimental groups. The control group received no intervention, while the experimental groups were subjected to vancomycin interventions for durations of 3, 4, 5, 6, and 7 days, respectively. The six groups of mice were housed in separate cages, labeled A, B, C, D, E, and F, to prevent data analysts from discerning the specific intervention details.

The correction factor (Km) is estimated by dividing the average body weight (kg) of a species by its body surface area (m²). The human Km is 37, and the animal Km is 3. To calculate the dose of vancomycin for animals, the animal dose (mg/kg) is based on the human vancomycin dose (mg/kg) using the formula: Animal dose (mg/kg) = Human dose (mg/kg) * (Human Km/Animal Km), which is used to convert to the vancomycin dose for mice (31). The recommended routine dose of vancomycin for adults is 20 mg/kg. Using the above formula, the vancomycin dose for mice is obtained as 12.3 mg/50g. Subsequently, based on the human administration method of four times a day, the mice were treated according to the aforementioned dosage.

At the designated time points, pancreatic tissue was collected for hematoxylin and eosin (HE) staining, and pancreatic injury was assessed using the Schmidt scoring criteria for acute pancreatitis (32). The animal experiments in this study were conducted in strict accordance with the ARRIVE guidelines(Animal Research: Reporting of *In Vivo* Experiments).

### Embedding of pancreatic tissue and hematoxylin and eosin(HE) staining

Pancreatic tissue is fixed using 4% paraformaldehyde and subsequently dehydrated with an ethanol solution. Xylene is then employed to clear the tissue before it is embedded in paraffin. Once the embedded tissue block hardens, thin sections are cut using a microtome to obtain tissue slices.

HE staining has been employed for over a century and is essential for identifying various tissue types and diagnosing diseases (33). Prior to HE staining, the paraffin is melted in an oven. Xylene is used to remove the paraffin from the sections, followed by a series of alcohol solutions of decreasing concentrations, and finally, the sections are washed with distilled water. After adequate dewaxing, hematoxylin stains the cell nuclei and nucleoli a bluish-purple, while eosin stains the cytoplasm red (34). The stained sections are dehydrated with pure alcohol and subsequently cleared with xylene. Finally, the cleared sections are covered with mounting medium and a cover slip to secure them.

### Acquisition of clinical data

This study conducted a retrospective analysis of the Medical Information Mart for Intensive Care IV 3.0(MIMIC-IV 3.0) database. The data in MIMIC-IV are sourced from the hospital’s electronic health record system and the ICU information system. MIMIC-IV 3.0 is a publicly available database that includes 364, 627 distinct patients treated at Beth Israel Deaconess Medical Center(BIDMC) in Boston between 2008 and 2022. These patients accounted for a total of 546, 028 admissions and 94, 458 unique ICU admissions (35). The Strengthening the Reporting of Observational Studies in Epidemiology(STROBE) checklist is in the Additional file 1 (32).

The treatment of acute pancreatitis primarily involves supportive care, and patients with severe acute pancreatitis should be referred to a tertiary care center with intensive care unit(ICU) facilities (36). Inclusion criteria: (a) Diagnosed with acute pancreatitis according to ICD-9(International Classification of Diseases, 9th Revision) or ICD-10(International Classification of Diseases, 10th Revision); (b) Age over 18 years; (c) Admitted to the ICU during the hospitalization. Exclusion criteria: (a) Unknown survival time and status during hospitalization or ICU stay; (b) Missing comorbidity information. For patients with multiple admissions, only information from the first hospitalization was collected. The extraction of data was conducted by the approved researcher(Wang Yongxiang).

### Patient characteristics and variables

Structured Query Language(SQL) was used to extract relevant medical information from the MIMIC-IV database. The following data were obtained: (1) Demographic information: age, gender, race, and body mass index(BMI); (2) Comorbidities determined by ICD-9 or ICD-10, including hypertension, type I diabetes, type II diabetes, heart failure, myocardial infarction, malignant tumors, chronic kidney disease, acute kidney failure, cirrhosis, hepatitis, tuberculosis, pneumonia, stroke, hyperlipidemia, and chronic bronchitis; (3) Laboratory indicators including complete blood count, blood biochemistry, blood gas analysis, coagulation, lipid profile, liver and kidney function, myocardial enzymes, and urinalysis; (4) Information on septicemia; (5) Antibiotic usage during the patient’s ICU stay, including cephalosporins, levofloxacin, meropenem, and vancomycin.

### Exposure and outcome

The use of vancomycin during ICU admission served as the exposure for patients. On one hand, independent risk factors for patient prognosis were used as the basis for subgroup analysis, while on the other hand, propensity scores were utilized to control for confounding factors, allowing for a comprehensive assessment of the impact of vancomycin on patients with severe pancreatitis.

The patients’ survival status served as the primary outcome measure for prognosis. To thoroughly evaluate the prognosis of patients with severe pancreatitis, the relationship was assessed from four aspects: prognosis during ICU admission, prognosis in the 28 days prior to ICU admission, prognosis during hospitalization, and prognosis in the 28 days prior to hospital admission. Patient follow-up continued until death, discharge, or the earliest time within 28 days after meeting the criteria.

### Data analysis

Continuous variables are expressed as mean ± standard deviation(SD). For comparisons of continuous variables, t-tests or analysis of variance(ANOVA) are used, or the Mann-Whitney U test or Kruskal-Wallis test are employed as necessary. Categorical variables are presented as counts or percentages(%), and differences between groups are compared using Pearson’s chi-square test or Fisher’s exact test.

Using the R package to perform propensity score matching, we assessed the distribution of covariates between the vancomycin group and the non-vancomycin group, thereby achieving a balance similar to randomization for confounding factors and reducing selection bias.

The Cox regression model was applied to assess the hazard ratio(HR) and its 95% confidence intervals(95% CIs) to determine the influence of the observed indicators on outcomes. Multivariate and univariate Cox regression models were performed using the R package to identify independent risk factors for patients with severe pancreatitis.

Additionally, a restricted cubic spline(RCS) with seven knots was utilized to illustrate the potential nonlinear relationship between vancomycin use and the outcomes.

Sensitivity analyses were conducted to determine how the relationship between vancomycin use and the occurrence of outcomes varied at different time points. Finally, subgroup analyses were performed based on the independent risk factors identified through Cox regression. All statistical analyses were conducted using the R statistical software package(R version 4.2.2), with a two-tailed P-value of <0.05 considered statistically significant.

## Results

### Vancomycin-induced pancreatic damage triggering pancreatitis

Among the side effects of vancomycin, 7.79% are classified as severe complications, 15.03% are related to urinary system adverse reactions, 5.86% to digestive system adverse reactions, 5.77% to respiratory system adverse reactions, 4.14% to circulatory system adverse reactions, 2.24% to skin adverse reactions, and 17.25% to blood profile or blood count adverse reactions (**Table 1**). Severe complications primarily involve organ failure in the lungs, kidneys, and liver. The urinary system issues mainly pertain to kidney function impairment. Adverse reactions related to the blood system primarily include eosinophilia, thrombocytopenia, and neutropenia. In terms of digestive system adverse reactions, 30.3% are liver injuries, 37.72% are diarrhea, and 25.24% are vomiting. In addition to these common side effects, 5.14% also report pancreatitis.

**Table 1:** Statistical analysis of vancomycin side effects.

Diseases	Category	Number of cases	Percentage
Severe complications	Multiple Organ Dysfunction Syndrome	921	0.020564003
	Renal Failure	1111	0.024806305
	Hepatic Failure	144	0.003215219
	Respiratory Failure	547	0.012213365
	Death	770	0.017192489
Urinary system	Acute Kidney Injury	4859	0.108491303
	Chronic Kidney Disease	66	0.001473642
	Renal Impairment	1809	0.040391185
Digestive system	Hepatic Necrosis	15	0.000334919
	Hepatitis Acute	27	0.000602854
	Liver Injury	796	0.017773014
	Diarrhoea	991	0.022126956
	Vomiting	663	0.014803403
	Pancreatitis	135	0.003014268
Respiratory system	Respiratory Tract Congestion	32	0.000714493
	Pneumonia	694	0.015495568
	Pulmonary Oedema	141	0.003148235
	Respiratory Acidosis	44	0.000982428
	Respiratory Disorder	106	0.002366758
	Respiratory Distress	1569	0.035032487
Circulatory system	Cardiac Arrest	445	0.009935919
	Cardiac Disorder	44	0.000982428
	Acute Myocardial Infarction	66	0.001473642
	Hypotension	1302	0.029070936
Skin	Rash	3139	0.070087302
	Pruritus	2345	0.052358943
	Toxic Epidermal Necrolysis	799	0.017839998
	Dermatitis	1723	0.038470985
	Urticaria	1090	0.024337419
	Erythema	949	0.021189184
Blood profile or blood counts	Eosinophilia	3510	0.078370956
	Neutropenia	924	0.020630987
	Thrombocytopenia	1443	0.032219171
	White Blood Cell Count Decreased	281	0.006274142
	Bilirubin Conjugated Increased	13	0.000290263
	Blood Creatinine Increased	1197	0.026726505
	Blood Urea Increased	356	0.007948735
Other	Other drug responses	9721	0.21704959

Next, we analyzed the relationship between vancomycin and acute pancreatitis. The results showed that among the 36, 037 samples using vancomycin, 35, 984 samples did not report acute pancreatitis as a side effect, while 53 samples reported acute pancreatitis. In comparison, among the 18, 253, 337 samples using other medications, 18, 234, 089 samples did not report pancreatitis as a side effect, while 19, 248 samples reported acute pancreatitis as a side effect. Subsequently, using the proportional reporting ratio(PRR) and the reporting odds ratio(ROR) for signal detection of adverse reactions, the ROR for vancomycin-induced acute pancreatitis was found to be 1.394(95% CI: 1.061315-1.820478), and the PRR was 1.394(95% CI: 1.061736-1.819756). This result suggests that vancomycin may induce pancreatic injury.

**Figure 2:**
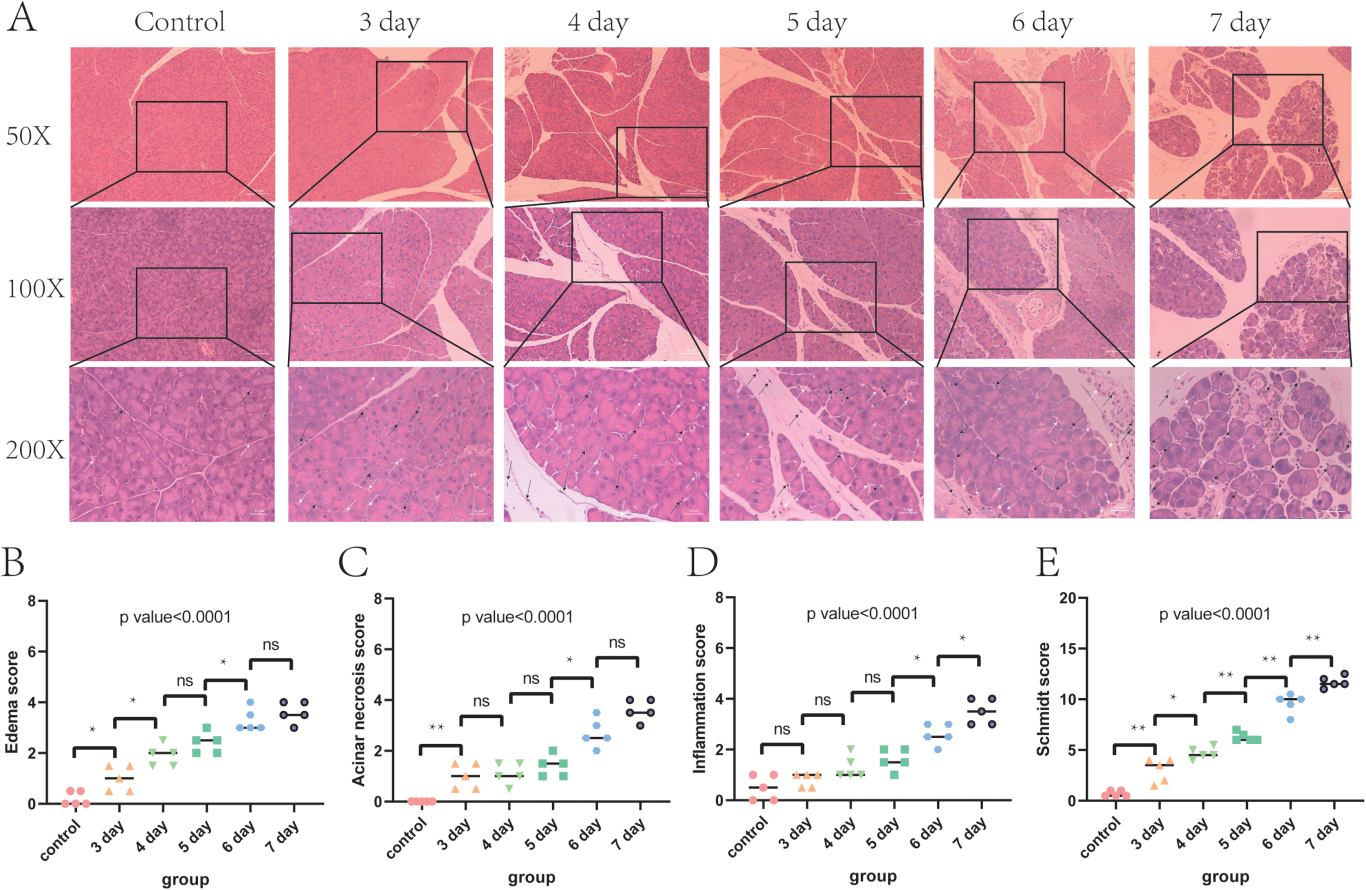
Vancomycin-induced pancreatitis(3, 4, 5, 6, and 7 days indicate the duration of vancomycin use). **(A)** HE staining results of pancreatic tissue (black arrows: white blood cells, white arrows: red blood cells). **(B)** Edema score of pancreatic tissue. **(C)** Acinar necrosis score of pancreatic tissue. **(D)** Inflammation score of pancreatic tissue. **(E)** Schmidt score of pancreatic tissue. *:=<0.05; **:=<0.01.

Consistent with it, animal experiments have demonstrated that the administration of vancomycin leads to a progressive deterioration of pancreatic damage. Between days 3 and 7, hematoxylin and eosin (HE) staining of pancreatic tissue reveals a temporal increase in edema and immune cell infiltration (**Figure 2A**). Compared to normal pancreatic tissue, pathological scores for edema, necrosis, and inflammation in the pancreatic tissues of vancomycin-treated mice were significantly elevated and positively correlated with the duration of vancomycin treatment (**Figures 2B-D**) (Detailed statistical information of group comparisons in **Supplementary Materials Tables S1**-**S3**). The acute pancreatitis Schmidt score results corroborate these findings (**Figure 2E**) (Detailed statistical information of group comparisons in **Supplementary Materials Table S4**).

Severe pancreatitis is often associated with organ failure across various systems and severe infection issues. Vancomycin is indispensable in controlling severe infections; however, the aforementioned information reveals the uncomfortable situation regarding the use of vancomycin in severe pancreatitis. It is crucial to clarify the impact of vancomycin on the prognosis of patients with severe pancreatitis.

### The relationship between vancomycin and the prognosis of patients with severe pancreatitis

**Figure 3:**
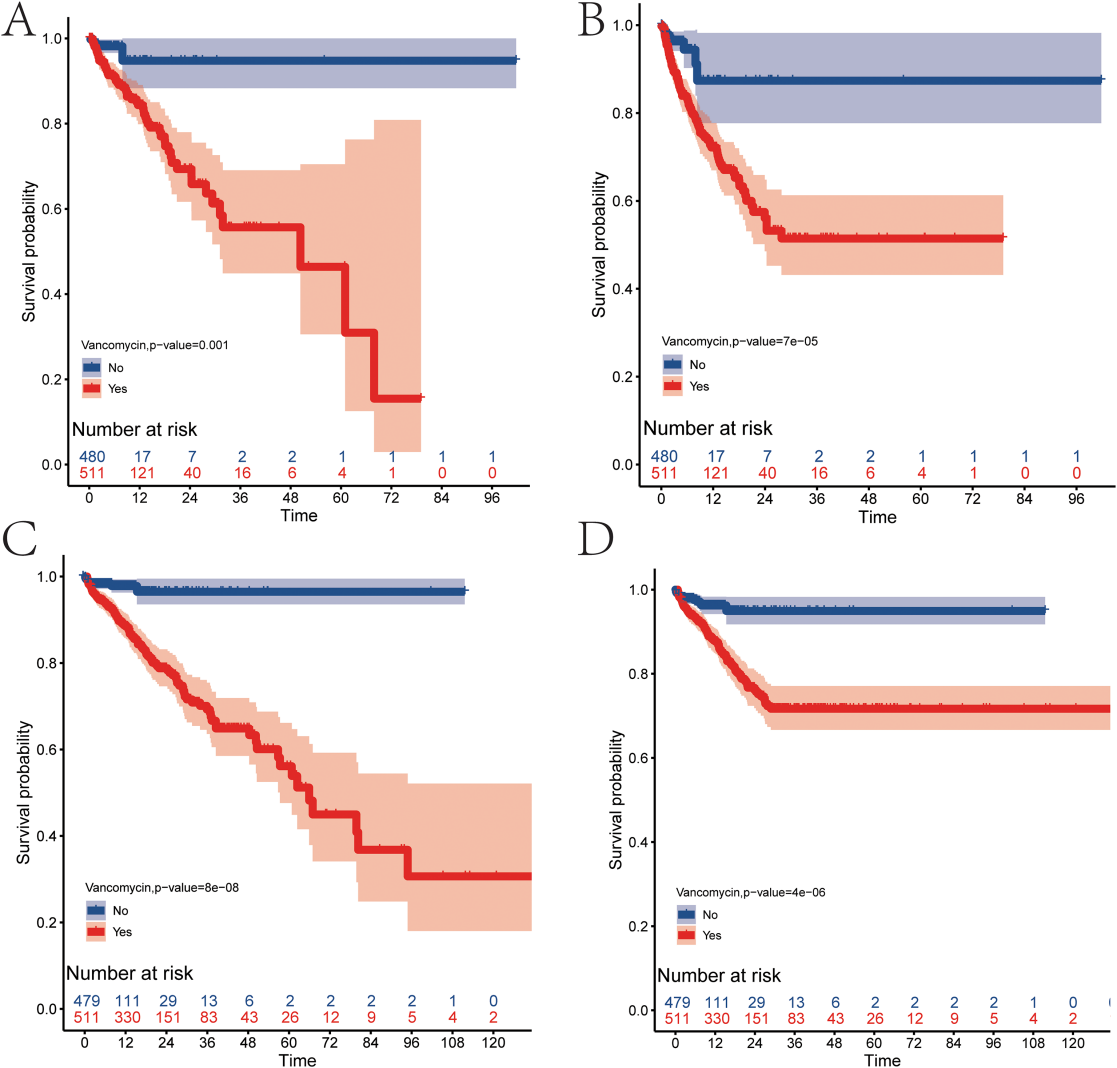
Survival analysis of patients with severe pancreatitis (blue curve represents patients not using vancomycin, red curve represents patients using vancomycin). **(A)** Survival curve during ICU admission. **(B)** Survival curve for the first 28 days of ICU admission. **(C)** Survival curve during hospitalization. **(D)** Survival curve for the first 28 days of hospitalization.

The use of vancomycin for patients with severe pancreatitis in the ICU increases the risk of mortality during their ICU stay(p=0.001) (**Figure 3A**). Severe pancreatitis patients experience rapid progression in the early stages due to a storm of inflammatory factors; thus, reducing early mortality rates can significantly improve the prognosis of severe pancreatitis. Vancomycin increases the risk of death in the 28 days prior to ICU admission(p=7e-05) (**Figure 3B**). After patients with severe pancreatitis are transferred out of the ICU, there remains a risk of death during the routine recovery process in the hospital. An analysis of overall survival data during hospitalization reveals that the use of vancomycin in the ICU also raises the overall mortality rate of patients with severe pancreatitis during their hospital stay(p=8e-08) (**Figure 3C**), and it increases the overall mortality rate for the first 28 days of hospitalization for these patients(p=4e-06) (**Figure 3D**).

The baseline data revealed that 511 patients with severe pancreatitis received vancomycin, while 480 did not. Statistical differences were observed between the two groups in terms of age(p=0.002) and vital signs, including systolic pressure(p<0.001), diastolic pressure(p<0.001), non-invasive mean blood pressure(p<0.001), and respiratory rate(p<0.001). Additionally, the incidence of sepsis(p<0.001), acute kidney injury(p<0.001), heart failure(p<0.001), chronic kidney disease(p=0.001), acute renal failure(p<0.001), liver cirrhosis(p=0.032), tuberculosis(p=0.01), and pneumonia(p<0.001) also demonstrated statistical differences between the two groups. Furthermore, laboratory indicators such as WBC(p<0.001), RBC(p=0.003), hemoglobin(p=0.001), RDW(p<0.001), hematocrit(p=0.01), albumin(p<0.001), potassium(p=0.01), total calcium(p=0.002), glucose(p=0.013), anion gap(p<0.001), lactate(p<0.001), total CO2(p=0.043), PT(p<0.001), PTT(p=0.016), INR(p<0.001), TG(p=0.02), total bilirubin(p<0.001), ALT(p=0.027), AST(p=0.001), urea nitrogen(p<0.001), and creatinine(p<0.001) also exhibited statistical differences (**Table 2**). The observed differences in baseline data may influence the prognostic role of vancomycin.

**Table 2:** Clinical baseline data of patients with severe pancreatitis.

Characteristics	Level	Non-use of vancomycin	Use of vancomycin	p	SMD
n		480	511		
age(mean(SD))		58.16(17.47)	61.49(16.64)	0.002	0.195
gender(%)	F	205(42.7)	217(42.5)	0.99	0.005
	M	275(57.3)	294(57.5)		
weight(mean(SD))		85.08(25.88)	85.76(23.64)	0.663	0.028
height(mean(SD))		169.31(6.91)	168.94(8.17)	0.439	0.049
Heartrate(mean(SD))		97.01(21.18)	99.72(22.67)	0.053	0.124
Systolic pressure(mean(SD))		132.73(24.40)	122.59(25.92)	<0.001	0.403
Diastolic pressure(mean(SD))		76.84(18.19)	70.28(19.75)	<0.001	0.223
Non-invasive mean blood pressure(mean(SD))		90.11(18.87)	82.53(20.39)	<0.001	0.386
Respiratory rate(mean(SD))		20.24(6.39)	21.84(6.84)	<0.001	0.242
Oxygen saturation%(mean(SD))		96.24(4.08)	95.95(4.18)	0.273	0.07
Temperature(°C)(mean(SD))		36.90(0.66)	36.90(0.93)	0.935	0.005
sepsis3(%)	No	283(59.0)	102(20.0)	<0.001	0.87
	Yes	197(41.0)	409(80.0)		
Acute kidney injury(%)	No	215(44.8)	105(20.5)	<0.001	0.535
	Yes	265(55.2)	406(79.5)		
Hypertension(%)	No	249(51.9)	293(57.3)	0.096	0.11
	Yes	231(48.1)	218(42.7)		
Type II diabetes(%)	No	347(72.3)	365(71.4)	0.817	0.019
	Yes	133(27.7)	146(28.6)		
Type I diabetes(%)	No	466(97.1)	502(98.2)	0.319	0.077
	Yes	14(2.9)	9(1.8)		
Heart failure(%)	No	415(86.5)	394(77.1)	<0.001	0.244
	Yes	65(13.5)	117(22.9)		
Myocardial infarction%)	No	470(97.9)	489(95.7)	0.072	0.127
	Yes	10(2.1)	22(4.3)		
Malignant tumor(%)	No	438(91.2)	458(89.6)	0.448	0.055
	Yes	42(8.8)	53(10.4)		
Chronic kidney disease(%)	No	430(89.6)	418(81.8)	0.001	0.224
	Yes	50(10.4)	93(18.2)		
Acute renal failure(%)	No	343(71.5)	201(39.3)	<0.001	0.683
	Yes	137(28.5)	310(60.7)		
Liver cirrhosis(%)	No	440(91.7)	446(87.3)	0.032	0.143
	Yes	40(8.3)	65(12.7)		
Hepatitis(%)	No	434(90.4)	453(88.6)	0.422	0.058
	Yes	46(9.6)	58(11.4)		
Tuberculosis(%)	No	471(98.1)	485(94.9)	0.01	0.176
	Yes	9(1.9)	26(5.1)		
Pneumonia(%)	No	424(88.3)	339(66.3)	<0.001	0.544
	Yes	56(11.7)	172(33.7)		
Stroke(%)	No	453(94.4)	475(93.0)	0.432	0.058
	Yes	27(5.6)	36(7.0)		
Hyperlipidemia(%)	No	346(72.1)	360(70.5)	0.619	0.036
	Yes	134(27.9)	151(29.5)		
Chronic bronchitis(%)	No	462(96.2)	482(94.3)	0.202	0.091
	Yes	18(3.8)	29(5.7)		
Levofloxacin(%)	No	465(96.9)	428(83.8)	<0.001	0.455
	Yes	15(3.1)	83(16.2)		
Cephalosporin(%)	No	409(85.2)	179(35.0)	<0.001	1.193
	Yes	71(14.8)	332(65.0)		
Ceftriaxone(%)	No	438(91.2)	385(75.3)	<0.001	0.437
	Yes	42(8.8)	126(24.7)		
Meropenem(%)	No	440(91.7)	367(71.8)	<0.001	0.532
	Yes	40(8.3)	144(28.2)		
WBC(K/Ul)(mean(SD))		12.35(7.17)	14.91(9.01)	<0.001	0.314
RBC(m/uL)(mean(SD))		3.75(0.72)	3.60(0.87)	0.003	0.19
Platelet count(K/Ul)(mean(SD))		215.85(127.39)	216.50(139.01)	0.939	0.005
hemoglobin(g/dL)(mean(SD))		11.37(2.17)	10.87(2.53)	0.001	0.211
RDW(%)(mean(SD))		14.86(1.97)	15.50(2.46)	<0.001	0.288
hematocrit(%)(mean(SD))		34.14(6.12)	33.01(7.49)	0.01	0.165
labalbumin(g/Dl)(mean(SD))		3.04(0.48)	2.82(0.57)	<0.001	0.423
sodium(mEq/L)(mean(SD))		138.06(4.95)	138.31(6.26)	0.497	0.043
potassium(mEq/L)(mean(SD))		4.06(0.75)	4.20(0.88)	0.01	0.164
Calcium total(mg/dL)(mean(SD))		8.05(0.98)	7.85(1.11)	0.002	0.193
chloride(mEq/L)(mean(SD))		103.86(6.62)	104.22(7.61)	0.44	0.049
Glucose (mg/dL)(mean(SD))		144.08(86.69)	160.91(122.28)	0.013	0.159
aniongap(mg/dL)(mean(SD))		15.61(4.81)	16.96(5.85)	<0.001	0.253
PH(mean(SD))		7.34(0.08)	7.33(0.12)	0.069	0.116
pco2(mmHg)(mean(SD))		40.60(8.18)	40.07(11.01)	0.387	0.055
po2(mmHg)(mean(SD))		105.52(59.42)	110.30(87.55)	0.319	0.064
lactate(mmol/L)(mean(SD))		2.30(1.41)	2.79(2.41)	<0.001	0.248
totalco2(mEq/L)(mean(SD))		23.23(4.82)	22.52(6.17)	0.043	0.129
freecalcium(mmol/L)(mean(SD))		1.07(0.09)	1.07(0.13)	0.273	0.07
pt(mean(SD))		15.68(6.77)	17.82(9.62)	<0.001	0.257
fibrinogen(mg/Dl)(mean(SD))		403.68(88.26)	404.58(161.28)	0.914	0.007
ptt(mean(SD))		33.78(15.42)	36.24(16.48)	0.016	0.154
INR(mean(SD))		1.43(0.68)	1.64(0.94)	<0.001	0.247
TG(mg/Dl)(mean(SD))		521.52(875.21)	413.52(558.93)	0.02	0.147
bilirubintotal(mg/dL)(mean(SD))		2.06(3.49)	3.13(5.43)	<0.001	0.234
ALT(IU/L)(mean(SD))		163.99(578.09)	270.55(893.49)	0.027	0.142
AST(IU/L)(mean(SD))		199.53(662.56)	527.82(2077.08)	0.001	0.213
Ureanitrogen(mg/Dl)(mean(SD))		21.44(20.01)	33.59(27.86)	<0.001	0.501
Creatinine(mg/Dl)(mean(SD))		1.33(1.50)	1.93(1.96)	<0.001	0.342

**Figure 4:**
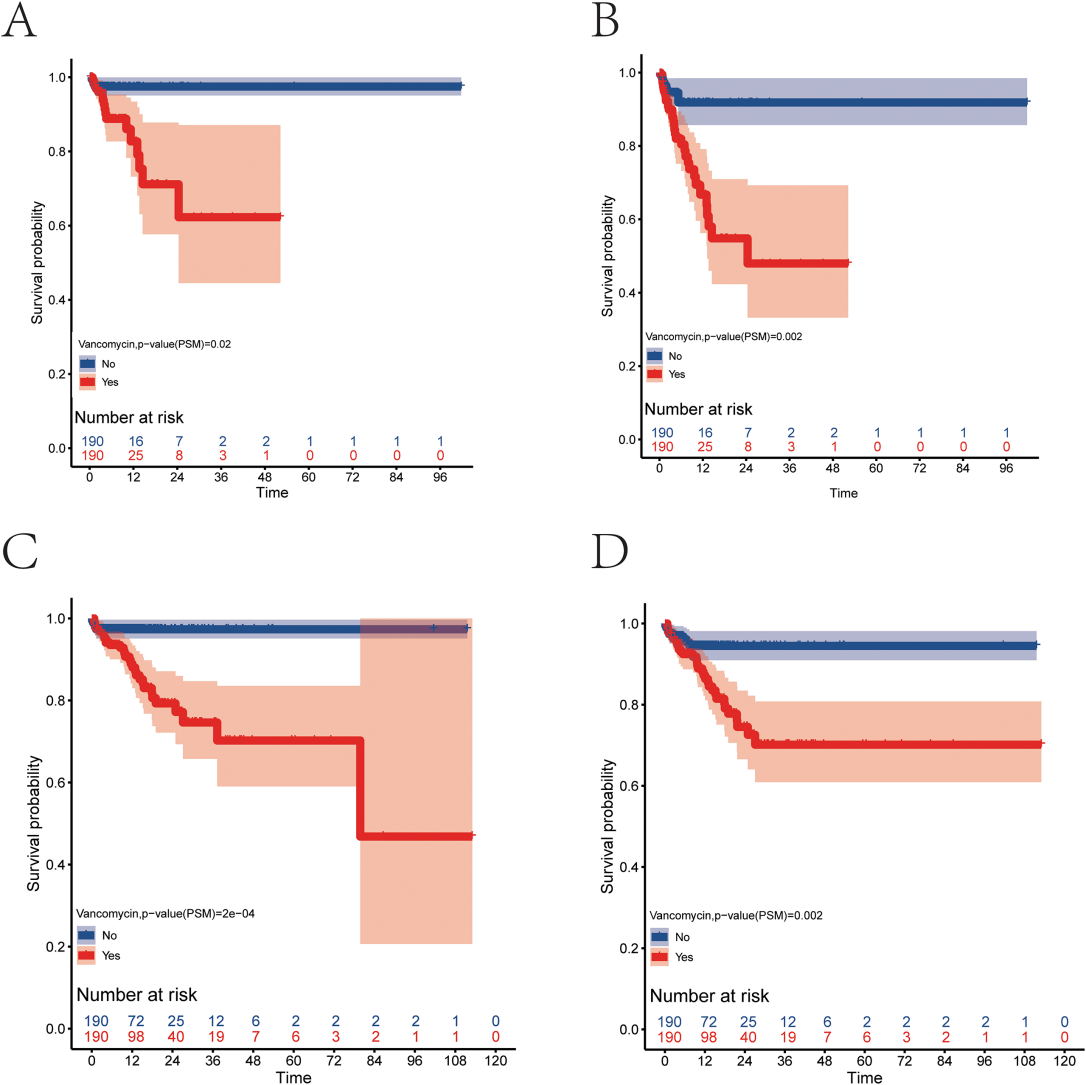
Survival analysis of patients with severe pancreatitis after propensity score matching (blue curve represents patients not using vancomycin, red curve represents patients using vancomycin). **(A)** Survival curve during ICU admission. **(B)** Survival curve for the first 28 days of ICU admission. **(C)** Survival curve during hospitalization. **(D)** Survival curve for the first 28 days of hospitalization.

To mitigate the influence of the aforementioned factors, this study employed propensity score matching to align clinical indicators between the two patient groups, effectively removing clinical differences and the impact of confounding factors in the prognosis analysis. A 1:1 matching was performed using propensity scores, resulting in 190 patients in both the vancomycin and non-vancomycin groups, with baseline differences between the groups eliminated post-matching (**Table 3**). The results indicated that the use of vancomycin for patients with severe pancreatitis in the ICU significantly increases the overall risk of mortality during their ICU stay(p=0.02) (**Figure 4A**) and within the 28 days prior to ICU admission(p=0.002) (**Figure 4B**). Additionally, an analysis of overall survival data during hospitalization revealed that the use of vancomycin in the ICU increases the overall mortality rate of patients with severe pancreatitis during their hospital stay(p=2e-04) (**Figure 4C**) and the overall mortality rate in the first 28 days of hospitalization(p=0.002) (**Figure 4D**).

**Table 3:** Clinical baseline data of patients with severe pancreatitis after propensity score matching.

Characteristics	Level	Non-use of vancomycin	Use of vancomycin	p	SMD
n		190	190		
age(mean(SD))		62.14(17.05)	59.70(16.65)	0.159	0.145
gender(%)	F	81(42.6)	85(44.7)	0.756	0.042
	M	109(57.4)	105(55.3)		
weight(mean(SD))		84.70(23.16)	84.73(23.10)	0.991	0.001
height(mean(SD))		168.79(7.05)	169.02(7.93)	0.764	0.031
Heart.rate(mean(SD))		100.09(21.28)	100.06(22.77)	0.987	0.002
systolic.pressure(mean(SD))		127.39(24.16)	127.28(25.35)	0.965	0.004
Diastolic pressure (mean(SD))		74.88(20.18)	72.67(18.13)	0.986	0.032
Non.invasive mean blood pressure (mean(SD))		85.92(19.76)	86.80(19.97)	0.665	0.045
Respiratory.rate(mean(SD))		21.20(7.25)	21.15(6.40)	0.94	0.008
Oxygen.saturation.(mean(SD))		95.85(5.12)	96.21(3.86)	0.442	0.079
Temperature(°C)(mean(SD))		36.91(0.68)	36.93(0.88)	0.805	0.025
sepsis(%)	No	57(30.0)	55(28.9)	0.91	0.023
	Yes	133(70.0)	135(71.1)		
Acute kidney injury(%)	No	52(27.4)	59(31.1)	0.498	0.081
	Yes	138(72.6)	131(68.9)		
Hypertension(%)	No	102(53.7)	97(51.1)	0.681	0.053
	Yes	88(46.3)	93(48.9)		
Type II diabetes(%)	No	145(76.3)	134(70.5)	0.246	0.131
	Yes	45(23.7)	56(29.5)		
Type I diabetes(%)	No	187(98.4)	185(97.4)	0.721	0.073
	Yes	3(1.6)	5(2.6)		
Heart failure(%)	No	150(78.9)	164(86.3)	0.078	0.195
	Yes	40(21.1)	26(13.7)		
Myocardial infarction(%)	No	185(97.4)	188(98.9)	0.445	0.118
	Yes	5(2.6)	2(1.1)		
Malignant tumor(%)	No	169(88.9)	172(90.5)	0.735	0.052
	Yes	21(11.1)	18(9.5)		
Chronic kidney disease(%)	No	161(84.7)	164(86.3)	0.771	0.045
	Yes	29(15.3)	26(13.7)		
Acute renal failure(%)	No	102(53.7)	101(53.2)	1	0.011
	Yes	88(46.3)	89(46.8)		
Liver cirrhosis(%)	No	172(90.5)	168(88.4)	0.616	0.069
	Yes	18(9.5)	22(11.6)		
Hepatitis(%)	No	170(89.5)	172(90.5)	0.864	0.035
	Yes	20(10.5)	18(9.5)		
Tuberculosis(%)	No	184(96.8)	183(96.3)	1	0.029
	Yes	6(3.2)	7(3.7)		
Pneumonia(%)	No	150(78.9)	151(79.5)	1	0.013
	Yes	40(21.1)	39(20.5)		
Stroke(%)	No	177(93.2)	177(93.2)	1	<0.001
	Yes	13(6.8)	13(6.8)		
Hyperlipidemia(%)	No	136(71.6)	138(72.6)	0.909	0.023
	Yes	54(28.4)	52(27.4)		
Chronic bronchitis(%)	No	178(93.7)	185(97.4)	0.137	0.179
	Yes	12(6.3)	5(2.6)		
Levofloxacin(%)	No	177(93.2)	180(94.7)	0.667	0.066
	Yes	13(6.8)	10(5.3)		
Cephalosporin(%)	No	123(64.7)	119(62.6)	0.749	0.044
	Yes	67(35.3)	71(37.4)		
Ceftriaxone(%)	No	152(80.0)	151(79.5)	1	0.013
	Yes	38(20.0)	39(20.5)		
Meropenem(%)	No	156(82.1)	153(80.5)	0.792	0.041
	Yes	34(17.9)	37(19.5)		
WBC(K/Ul)(mean(SD))		14.08(8.94)	14.66(8.99)	0.526	0.065
RBC(m/uL)(mean(SD))		3.64(0.76)	3.69(0.87)	0.561	0.06
Platelet count(K/Ul) (mean(SD))		232.10(149.26)	218.71(131.28)	0.354	0.095
hemoglobin(g/dL)(mean(SD))		11.05(2.37)	11.19(2.54)	0.577	0.057
RDW(%)(mean(SD))		15.05(2.17)	14.95(1.88)	0.62	0.051
hematocrit(%)(mean(SD))		33.38(6.80)	33.79(7.59)	0.581	0.057
labalbumin(g/Dl)(mean(SD))		2.92(0.48)	2.90(0.55)	0.682	0.042
sodium(mEq/L)(mean(SD))		138.01(5.27)	138.08(6.03)	0.892	0.014
potassium(mEq/L)(mean(SD))		4.11(0.73)	4.09(0.84)	0.795	0.027
Calcium total(mg/dL)(mean(SD))		7.83(1.13)	7.89(1.21)	0.617	0.051
chloride(mEq/L)(mean(SD))		103.87(7.25)	103.71(7.17)	0.82	0.023
glucose(mg/dL)(mean(SD))		146.55(95.78)	156.17(106.53)	0.355	0.095
aniongap(mg/dL)(mean(SD))		16.00(5.23)	16.22(5.78)	0.696	0.04
PH(mean(SD))		7.34(0.09)	7.34(0.11)	0.584	0.056
pco2(mmHg)(mean(SD))		40.72(8.72)	41.29(11.41)	0.579	0.057
po2(mmHg)(mean(SD))		108.24(67.70)	110.68(87.61)	0.761	0.031
lactate(mmol/L)(mean(SD))		2.55(1.96)	2.49(1.61)	0.717	0.037
totalco2(mEq/L)(mean(SD))		23.24(5.66)	23.30(6.13)	0.917	0.011
Free calcium(mmol/L)(mean(SD))		1.06(0.09)	1.06(0.13)	0.527	0.065
pt(mean(SD))		17.46(9.22)	16.73(7.27)	0.392	0.088
fibrinogen(mg/Dl)(mean(SD))		404.09(121.61)	400.31(139.62)	0.778	0.029
ptt(mean(SD))		34.59(15.86)	34.53(14.19)	0.972	0.004
INR(mean(SD))		1.61(0.91)	1.53(0.73)	0.385	0.089
TG(mg/Dl)(mean(SD))		424.34(593.72)	393.35(439.28)	0.563	0.059
Bilirubin total(mg/dL)(mean(SD))		2.58(4.93)	2.65(4.36)	0.891	0.014
ALT(IU/L)(mean(SD))		219.93(881.17)	222.04(716.46)	0.98	0.003
AST(IU/L)(mean(SD))		274.21(1010.27)	420.69(1910.47)	0.351	0.096
Ureanitrogen(mg/Dl)(mean(SD))		26.68(22.54)	27.69(25.73)	0.686	0.042
Creatinine(mg/Dl)(mean(SD))		1.64(1.77)	1.77(2.29)	0.534	0.064

### Subgroup analysis and mediation analysis of vancomycin on overall prognosis in the ICU

**Figure 5:**
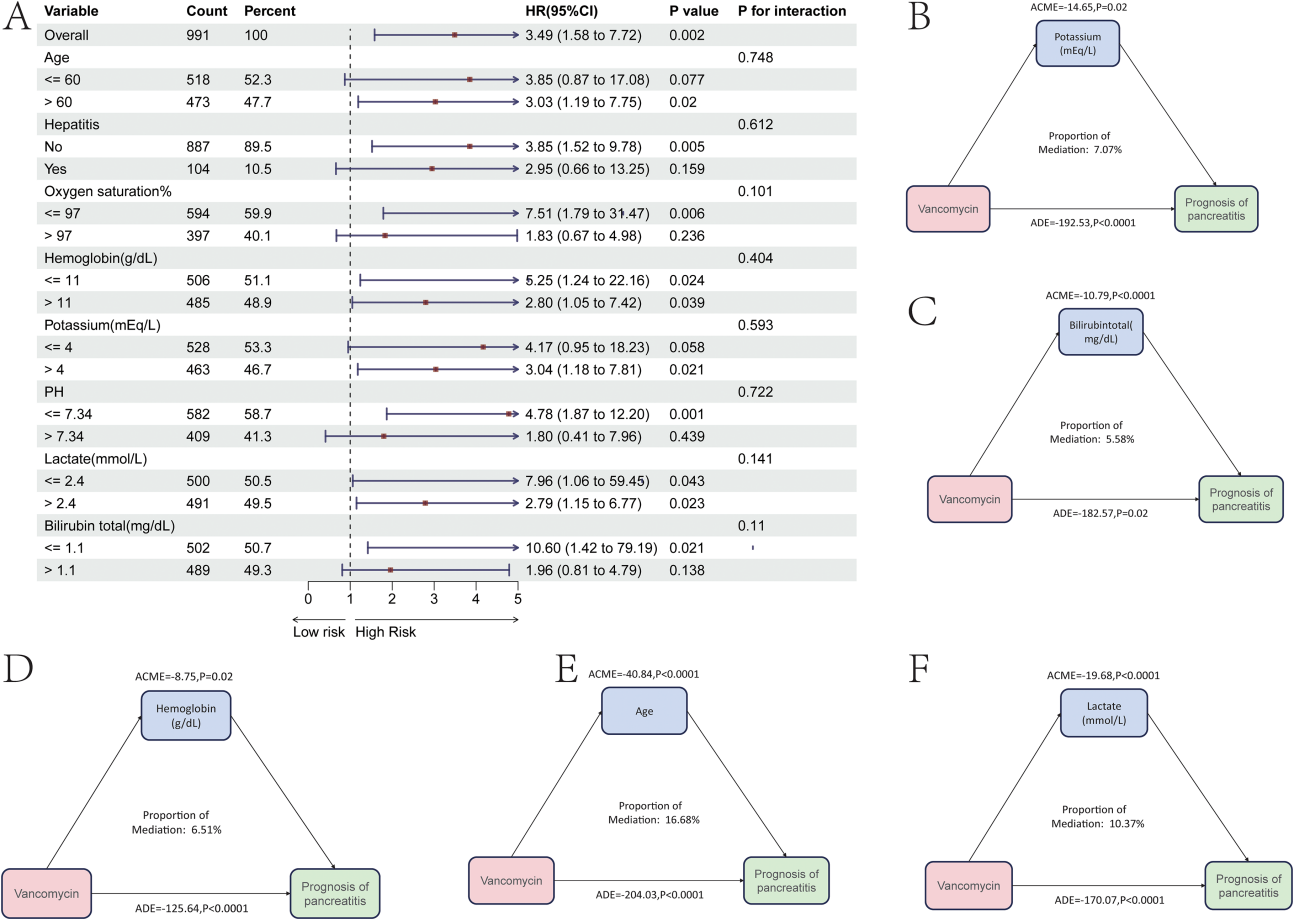
Subgroup and mediation analysis of the prognostic impact of vancomycin on patients with severe pancreatitis during ICU admission. **(A)** Prognostic analysis of clinical subgroups related to vancomycin use during ICU admission. **(B-F)** Weights of various independent risk factors mediating the prognosis affected by vancomycin in patients with severe pancreatitis during ICU admission.

Through univariate and multivariate COX regression analyses, independent risk factors affecting the prognosis of patients with severe pancreatitis during their ICU stay were identified. The results showed that, in addition to vancomycin, factors such as age, the presence of hepatitis, oxygen saturation, hemoglobin levels, potassium concentration, pH value, lactate concentration, and total bilirubin concentration were independent risk factors (**Table 4**). Subsequently, subgroup analyses were conducted based on these independent risk factors, with continuous variables grouped according to their median. The results indicated that for severe pancreatitis patients older than 60 years, the risk of mortality increased after using vancomycin(HR = 3.03, P = 0.02). For patients with an oxygen saturation level below 97%, the risk of mortality also increased after vancomycin use(HR = 7.51, P = 0.005). Additionally, patients with a potassium concentration greater than 4 mEq/L(HR = 3.04, P = 0.021) or a pH greater than 7.34(HR = 4.78, P = 0.001) also experienced an increased risk of mortality after using vancomycin (**Figure 5A**). However, severe pancreatitis patients without concurrent hepatitis showed an increased risk of mortality after using vancomycin(HR = 3.85, P = 0.005). Correspondingly, patients with a total bilirubin level below 1.1 mg/dL also exhibited an increased mortality risk after using vancomycin(HR = 10.6, P = 0.021). This may be due to the fact that vancomycin can cause a certain degree of liver damage during treatment, thereby significantly affecting patients without pre-existing liver injury. In contrast, for those with existing liver injury, vancomycin does not further increase liver damage, and thus, there is no significant change in mortality risk for this group of patients.

**Table 4:** Univariate and multivariate COX regression of overall prognosis in the ICU.

Characteristics	Univariate			Multivariate		
	HR	CI	p	HR	CI	P
age	1.04	1.03 - 1.06	0	1.06	1.04 - 1.08	0
Heart failure						
No	reference			reference		
Yes	1.97	1.17 - 3.31	0.01	1.5	0.79 - 2.85	0.2102
Acute renal failure						
No	reference			reference		
Yes	2.78	1.51 - 5.13	0.001	1.13	0.57 - 2.25	0.7211
Liver cirrhosis						
No	reference			reference		
Yes	2.2	1.28 - 3.78	0.004	1.35	0.58 - 3.18	0.4883
Hepatitis						
No	reference			reference		
Yes	2.24	1.27 - 3.95	0.006	4.51	2.2 - 9.26	0
Vancomycin						
No	reference			reference		
Yes	3.49	1.58 - 7.72	0.002	2.39	1.07 - 5.31	0.0328
Systolic pressure	0.99	0.98 - 1	0.006	1	0.99 - 1.01	0.7993
Oxygen saturation	0.95	0.93 - 0.98	0	0.97	0.94 - 0.99	0.02
Temperature(°C)	0.67	0.55 - 0.82	0	1.06	0.8 - 1.4	0.6709
WBC(K/Ul)	0.71	0.55 - 0.93	0.014	1.14	0.49 - 2.63	0.7592
hemoglobin(g/dL)	0.88	0.8 - 0.97	0.008	0.89	0.81 - 0.99	0.027
RDW(%)	1.13	1.05 - 1.21	0.001	1.03	0.92 - 1.15	0.6232
potassium(mEq/L)	1.47	1.19 - 1.82	0	1.26	0.97 - 1.63	0.0784
aniongap(mg/dL)	1.08	1.05 - 1.11	0	1	0.94 - 1.06	0.9412
PH	0.02	0 - 0.11	0	0.05	0 - 0.5	0.0105
lactate(mmol/L)	1.19	1.13 - 1.26	0	1.08	1.01 - 1.15	0.0275
totalco2(mEq/L)	0.92	0.89 - 0.96	0	0.98	0.93 - 1.04	0.5263
fibrinogen(mg/Dl)	1.03	1.01-1.07	0.02	1.01	0.98-1.05	0.4929
ptt	1.01	1.01 - 1.02	0	1	0.99 - 1.01	0.7653
bilirubintotal(mg/dL)	1.05	1.02 - 1.07	0	1.03	1 - 1.06	0.0768
Ureanitrogen(mg/Dl)	1.02	1.01 - 1.02	0	1	0.99 - 1.01	0.543
Creatinine(mg/Dl)	1.15	1.06 - 1.25	0.001	0.99	0.84 - 1.17	0.8929

Further mediation analysis revealed that the age, hemoglobin concentration, potassium concentration, lactate concentration, and total bilirubin concentration of patients with severe pancreatitis mediated the mortality risk associated with vancomycin. The mediation effect of potassium in the relationship between vancomycin and the prognosis of severe pancreatitis accounted for only 7.07% of the total effect (**Figure 5B**). The mediation effect of total bilirubin in the relationship between vancomycin and the prognosis of severe pancreatitis accounted for only 5.58% of the total effect (**Figure 5C**). The mediation effect of hemoglobin in this relationship accounted for only 6.51% of the total effect (**Figure 5D**). The mediation effect of age in the relationship between vancomycin and the prognosis of severe pancreatitis accounted for only 16.68% of the total effect (**Figure 5E**). The mediation effect of lactate in this relationship accounted for only 10.37% of the total effect (**Figure 5F**).

### Subgroup analysis and mediation analysis of vancomycin on prognosis in the 28 days prior to ICU admission

**Figure 6:**
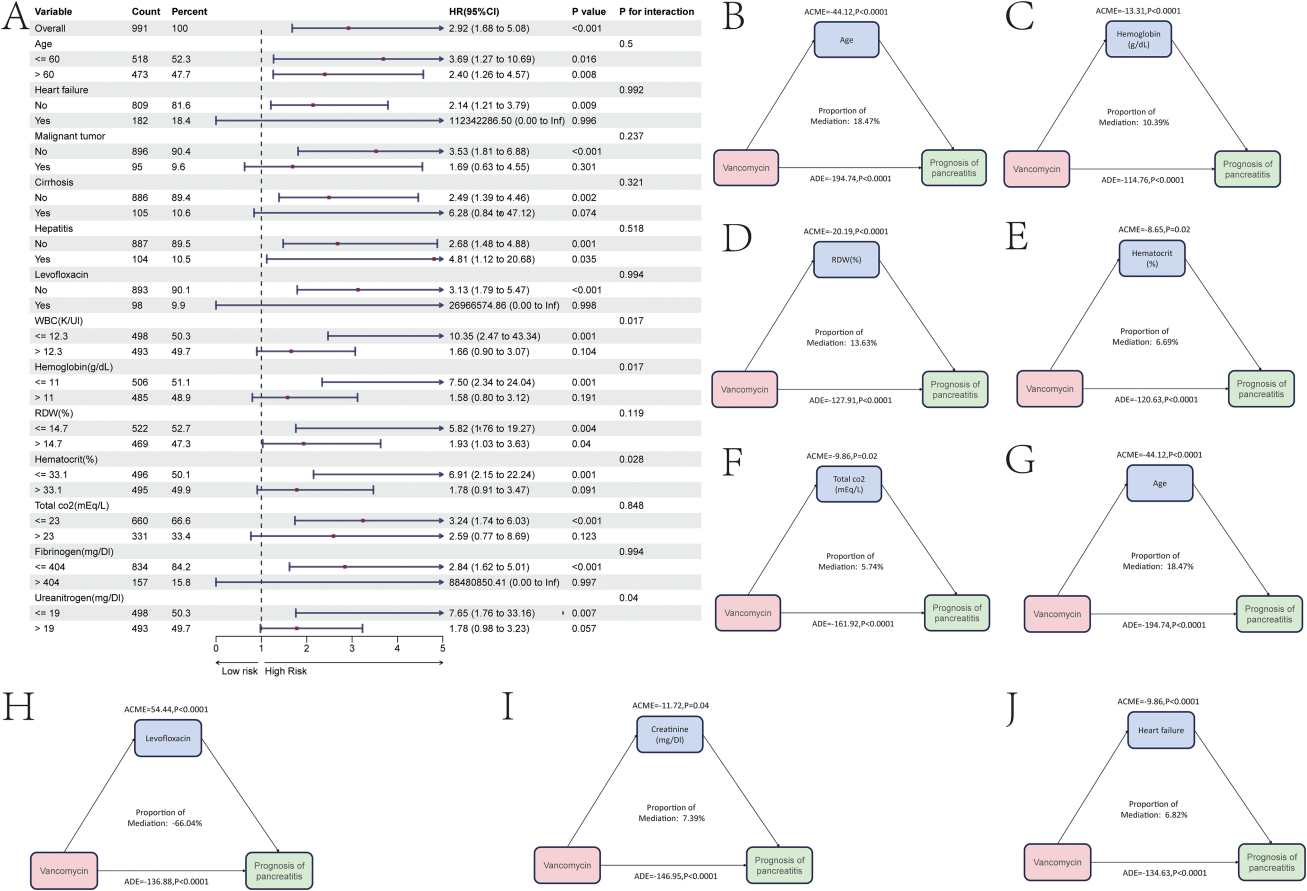
Subgroup and mediation analysis of the prognostic impact of vancomycin on patients with svere pancreatitis in the 28 days prior to ICU admission. **(A)** Prognostic analysis of clinical subgroups related to vancomycin use in the 28 days prior to ICU admission. **(B-J)** Weights of various independent risk factors mediating the prognosis affected by vancomycin in patients with severe pancreatitis during the 28 days prior to ICU admission.

Through univariate and multivariate COX regression analyses, it was found that the independent risk factors for prognosis in the 28 days prior to ICU admission were more numerous than those for overall prognosis in the ICU. In addition to vancomycin, the independent risk factors for severe pancreatitis included age, heart failure, tumors, liver fibrosis and hepatitis status, whether levofloxacin was used during ICU admission, as well as the number of red blood cells, hemoglobin levels, red cell distribution width, hematocrit, CO2 concentration, fibrinogen, and urine protein (**Table 5**). Subgroup analysis based on these independent risk factors revealed that severe pancreatitis patients with heart failure, malignancy, or cirrhosis did not have an increased risk of death after using vancomycin. However, severe pancreatitis patients without heart failure showed an increased mortality risk after using vancomycin(HR = 2.14, p=0.009), those without a tumor showed an increased risk(HR = 3.53, p<0.001), and those without cirrhosis also showed an increased risk(HR = 2.49, p=0.002). Severe pancreatitis patients who had received levofloxacin did not experience an increased risk of death when using vancomycin, whereas patients who did not use vancomycin showed an increased mortality risk after using it(HR = 3.13, p<0.001).The condition of red blood cells in severe pancreatitis patients also impacted the prognosis mediated by vancomycin. Patients with a red blood cell count of 12.3K/UI or less(HR = 3.13, p<0.001), a hemoglobin level of 11 g/dL or less(HR = 7.5, p=0.001), a red cell distribution width of 14.7% or less(HR = 5.82, p=0.004), or a hematocrit of 33.1% or less(HR = 6.91, p=0.001) all faced increased mortality risk after using vancomycin. Additionally, severe pancreatitis patients with a CO2 concentration of 23 mEq/L or less(HR = 3.24, p<0.001), prothrombin of 404 mg/DL or less(HR = 2.84, p<0.001), or urine protein of 19 mg/DL or less(HR = 7.65, p=0.007) also showed an increased risk of death after using vancomycin (**Figure 6A**).

**Table 5:** Univariate and multivariate COX regression of prognosis in the 28 days prior to ICU admission.

Characteristics	Univariate			Multivariate		
	HR	CI	p	HR	CI	P
age	1.04	1.03 - 1.06	0	1.05	1.03 - 1.07	0
Heart failure						
No	reference			reference		
Yes	2	1.35 - 2.95	0	1.68	1.05 - 2.71	0.0313
Malignant tumor						
No	reference			reference		
Yes	2.67	1.72 - 4.14	0	2.2	1.36 - 3.57	0.0014
Chronic kidney disease						
No	reference			reference		
Yes	1.72	1.12 - 2.65	0.01	0.94	0.57 - 1.55	0.8023
Acute renal failure						
No	reference			reference		
Yes	2.37	1.53 - 3.67	0	1.09	0.66 - 1.79	0.7428
Liver cirrhosis						
No	reference			reference		
Yes	2.41	1.61 - 3.63	0	2.17	1.16 - 4.05	0.0153
Hepatitis						
No	reference			reference		
Yes	2.02	1.29 - 3.15	0	3.21	1.71 - 6.04	0.0003
Levofloxacin						
No	reference			reference		
Yes	0.52	0.27 - 0.99	0.05	0.47	0.23 - 0.93	0.0308
Vancomycin						
No	reference			reference		
Yes	2.92	1.68 - 5.08	0	2.01	1.11 - 3.63	0.0208
weight	0.99	0.98 - 1	0.02	1	0.99 - 1.01	0.3666
Heart rate	0.99	0.98 - 1	0.04	1	0.99 - 1.01	0.5325
Systolic pressure	0.99	0.98 - 1	0	1	0.99 - 1.01	0.6447
Non invasive.mean. Blood pressure	0.99	0.98 - 1	0.01	1.01	0.99 - 1.02	0.5059
Temperature(oC)	0.68	0.58 - 0.79	0	0.92	0.74 - 1.13	0.4287
WBC(K/Ul)	1.02	1 - 1.04	0.01	1.02	1 - 1.03	0.0885
RBC(m/uL)	0.7	0.56 - 0.87	0	1.02	1 - 1.03	0.0885
hemoglobin(g/dL)	0.86	0.8 - 0.93	0	0.58	0.43 - 0.78	0.0003
RDW(%)	1.16	1.11 - 1.22	0	1.08	1 - 1.15	0.0388
hematocrit(%)	0.96	0.94 - 0.99	0	1.2	1.09 - 1.32	0.0002
potassium(mEq/L)	1.28	1.06 - 1.53	0.01	1.03	0.82 - 1.31	0.7862
aniongap(mg/dL)	1.06	1.03 - 1.09	0	0.98	0.93 - 1.03	0.4296
PH	0.16	0.04 - 0.69	0.01	0.79	0.08 - 7.42	0.8374
lactate(mmol/L)	1.16	1.11 - 1.21	0	1.04	0.96 - 1.13	0.3029
totalco2(mEq/L)	0.95	0.92 - 0.97	0	0.95	0.92 - 0.98	0.0008
fibrinogen(mg/Dl)	1.05	1.01-1.08	0	1.02	1.01-1.05	0.041
ptt	1.01	1 - 1.02	0.01	1	0.99 - 1.01	0.5087
bilirubintotal(mg/dL)	1.04	1.02 - 1.06	0	1.01	0.98 - 1.04	0.4067
Ureanitrogen(mg/Dl)	1.01	1.01 - 1.02	0	1.01	1 - 1.01	0.0419
Creatinine(mg/Dl)	1.11	1.03 - 1.2	0	0.97	0.85 - 1.1	0.6254

Further mediation analysis revealed that factors such as age, hemoglobin concentration, red cell distribution width, hematocrit, CO2 concentration, the use of levofloxacin, and heart failure mediated the mortality risk associated with vancomycin in patients with severe pancreatitis. The mediation effect of age in the relationship between vancomycin and prognosis in severe pancreatitis accounted for only 18.47% of the total effect (**Figure 6B**). The mediation effect of hemoglobin concentration in this relationship accounted for only 10.39% of the total effect (**Figure 6C**). The mediation effect of red cell distribution width accounted for only 13.63% of the total effect (**Figure 6D**). The mediation effect of hematocrit accounted for only 6.69% of the total effect (**Figure 6E**). The mediation effect of total CO2 concentration accounted for only 5.74% of the total effect (**Figure 6F**). The mediation effect of ureanitrogen accounted for only 20.07% of the total effect (**Figure 6G**). The mediation effect of levofloxacin in this relationship was also noted to be -66.04% (**Figure 6H**). The mediation effect of creatinine accounted for only 7.39% of the total effect (**Figure 6I**). The mediation effect of heart failure accounted for only 6.82% of the total effect (**Figure 6J**).

### The relationship between vancomycin dosage and mortality risk in severe pancreatitis

**Figure 7:**
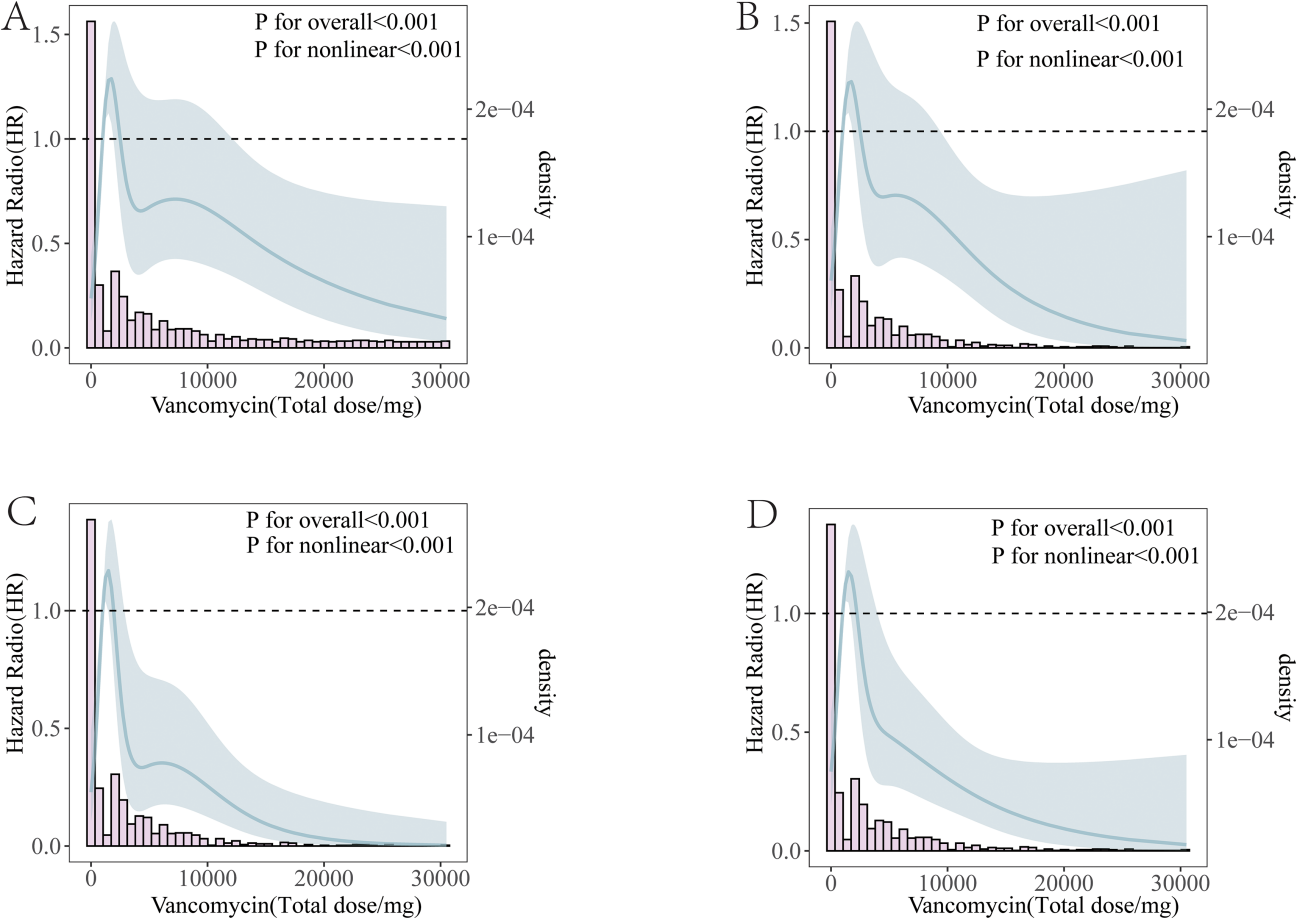
Relationship Between Mortality Risk and Vancomycin Dosage in Patients with Severe Pancreatitis. **(A)** Relationship between mortality risk and vancomycin dosage during hospitalization in patients with severe pancreatitis. **(B)** Relationship between mortality risk and vancomycin dosage in the 28 days prior to hospitalization in patients with severe pancreatitis. **(C)** Relationship between mortality risk and vancomycin dosage during ICU admission in patients with severe pancreatitis. **(D)** Relationship between mortality risk and vancomycin dosage in the 28 days prior to ICU admission in patients with severe pancreatitis.

RCS analysis revealed that the total dosage of vancomycin exhibited a “U-shaped” relationship with the mortality risk in severe pancreatitis. The RCS cubic plot for prognosis during hospitalization for severe pancreatitis showed that the mortality risk initially increased with increasing doses of vancomycin and then decreased as the dosage continued to increase. The total dosages of 1000 mg and 2000 mg for vancomycin served as critical points for distinguishing the mortality risk of patients with severe pancreatitis during hospitalization (**Figure 7A**). Similarly, the RCS cubic plot of prognosis during the first 28 days of hospitalization for severe pancreatitis also indicated that the mortality risk first increased with vancomycin dosage and then decreased as the dosage continued to rise, with the total dosages of 1000 mg and 2500 mg serving as distinguishing points for the mortality risk in the first 28 days of hospitalization (**Figure 7B**).

The RCS cubic plot for prognosis during ICU admission for severe pancreatitis showed that the mortality risk initially increased with increasing doses of vancomycin and then decreased as the dosage continued to rise. This trend is consistent with the distinguishing dosage points observed during hospitalization, where the total dosages of 1000 mg and 2000 mg of vancomycin also served as critical points for mortality risk in patients with severe pancreatitis during their ICU stay (**Figure 7C**). Similarly, the RCS cubic plot for prognosis during the first 28 days of ICU admission for severe pancreatitis also indicated that the mortality risk increased with vancomycin dosage initially and then decreased as the dosage continued to rise, with the total dosages of 1000 mg and 2200 mg serving as distinguishing points for the mortality risk during the first 28 days in the ICU (**Figure 7D**).

Based on these cutoff values, vancomycin dosage ranges were categorized into three groups: low-dose, medium-dose, and high-dose. Cox regression analysis indicated that the lowest mortality risk occurred in the low-dose group, followed by the high-dose group, while the medium-dose group exhibited the highest risk during hospitalization and in the 28 days preceding hospitalization. Furthermore, after ICU admission and during the days leading up to admission, no statistically significant difference in mortality risk was observed between the low-dose and high-dose groups; however, the medium-dose group showed a significantly higher risk compared to the other two groups (**Table 6**).

**Table 6:** The effect of different doses of vancomycin on the prognosis of patients with severe pancreatitis.

Characteristics	Group	HR	CI5	CI95	p
Hosp_day	<1000mg	reference			
	>2000mg	2.67	1.45	4.92	0.002
	1000-2000mg	4.26	2.21	8.23	0
ICU	<1000mg	reference			
	>2000mg	1.33	0.62	2.86	0.47
	1000-2000mg	3.41	1.52	7.63	0.003
Hosp_28day	<1000mg	reference			
	>2500mg	2.06	1.18	3.59	0.011
	1000-2500mg	3.51	1.96	6.3	0
ICU_28day	<1000mg	reference			
	>2200mg	1.31	0.75	2.29	0.336
	1000-2200mg	3.06	1.72	5.47	0

## Discussion

Although vancomycin targets the microbial flora associated with secondary infections in pancreatitis and helps in controlling infections, the results of this study suggest that patients with severe pancreatitis who use vancomycin may have poorer prognoses. This raises a thought-provoking issue. Globally, the problem of antibiotic resistance is becoming increasingly severe, leading healthcare institutions to adopt a more conservative and cautious approach to the use of antibiotics in clinical practice (37). However, acute pancreatitis progresses rapidly, leaving healthcare providers with a limited decision-making time window. If the intervention with vancomycin is missed, it may not only fail to control the infection but also fail to alleviate the persistent damage caused by the infection. The results of this study show that among the vast majority of patients included in the MIMIC database, regardless of whether or not they developed septicemia, over 60% of patients received a total dose of vancomycin less than 5000 mg.

Our team has also demonstrated that vancomycin can damage pancreatic tissue and induce acute pancreatitis in mice in a dose-dependent manner. These results suggest that vancomycin may also cause damage to human pancreatic tissue. Therefore, we hope that future researchers and clinical practitioners will increase their attention to this issue. However, how vancomycin causes damage to pancreatic tissue is an intriguing question. Previous studies have shown that vancomycin can lead to the apoptosis of endothelial cells and result in vascular damage (38). The use of vancomycin can lead to an increase in reactive oxygen species (ROS) and oxidative stress (39). The production of these substances may contribute to the mechanisms through which vancomycin causes harm to the human body. Vascular damage can lead to injury in various organs, often manifested as liver and kidney toxicity during clinical application. In conjunction with this study, this may be the mechanism by which vancomycin damages pancreatic tissue and triggers acute pancreatitis.

As the dosage of vancomycin increased, the mortality risk for patients with severe pancreatitis initially increased and then decreased; however, the mortality risk for patients receiving a total dose of less than 5000 mg remained higher than that of patients who did not use vancomycin. These findings indicate that the current clinical use of vancomycin for severe pancreatitis patients tends to be cautious. Nevertheless, infections are closely related to organ dysfunction in acute pancreatitis. Therefore, the rational use of vancomycin to prevent or control infections in severe pancreatitis can reduce the incidence of organ failure (40, 41), which is beneficial for improving patient prognosis. Thus, identifying the clinical factors that influence vancomycin-related prognosis is crucial for improving the ultimate outcomes of patients with severe pancreatitis. Vancomycin must maintain a continuous effect to effectively control infections; therefore, short-term use of this antibiotic is inadequate for infection management and may increase mortality due to its hepatotoxic and nephrotoxic effects (42). As the duration of treatment and cumulative dosage increase, vancomycin gradually exerts its antimicrobial properties, leading to a reduction in infection-related mortality, which progressively offsets the mortality associated with its hepatotoxicity and nephrotoxicity. Consequently, the overall mortality rate initially rises before subsequently declining. In patients with severe pancreatitis, the cumulative dosage of vancomycin displays a U-shaped relationship with patient mortality.

Advanced age is a risk factor for pancreatitis, as older patients have a higher likelihood of organ failure and other complications (43, 44). Organ dysfunction is the leading cause of mortality in acute pancreatitis (45–47). In cases of pancreatitis, the respiratory and renal systems are the most commonly affected (48). The mortality rates for patients with severe acute pancreatitis who experience respiratory, renal, and liver failure are 43%, 63%, and 83%, respectively (49). Although the impacts of respiratory failure and renal failure are similar, respiratory failure remains the most common type of organ dysfunction (41, 50, 51). The results of this study also confirm that advanced age is a risk factor for severe pancreatitis, and that patients over the age of 60 who use vancomycin have an increased risk of mortality. This may be related to alterations in the normal pharmacokinetic processes of vancomycin following organ failure.

This study found that when oxygen saturation is below 97% or total CO2 levels are less than 23, the use of vancomycin increases the mortality risk in patients with severe pancreatitis.This result suggests that special caution is needed when using vancomycin to control infections in patients with severe pancreatitis who experience respiratory failure.Previous studies have indicated that vancomycin can induce mild hepatic injury (52). However, our findings show that the use of vancomycin in patients with severe pancreatitis who also have hepatitis does not increase the mortality risk. Conversely, the mortality risk increases in patients with severe pancreatitis without concurrent hepatitis after using vancomycin, and the risk also increases for patients with total bilirubin levels below 1 mg/dL.This result is quite intriguing, and the specific reasons remain to be investigated further. Nevertheless, it suggests that in patients with severe pancreatitis who experience liver failure, the use of vancomycin to control infections does not increase mortality risk.

Previous studies have indicated that blood urea nitrogen, creatinine, white blood cell count, heart rate, procalcitonin, and albumin can serve as risk factors for patients with pancreatitis (53, 54). Vancomycin is associated with an increased incidence of acute kidney injury(AKI), which can range from 5% to 35% (55). Some researchers have pointed out that early blood urea nitrogen levels in patients with acute pancreatitis can serve as prognostic predictors, and that serum creatinine levels exceeding 2 mg/dL can be used to identify severely affected or clinically worsening patients (56). Additionally, proteinuria is closely related to severe pancreatitis and can help differentiate between severe acute pancreatitis(SAP) and mild acute pancreatitis(MAP) (57). This study found an interaction between vancomycin, creatinine, and protein levels in patients with severe pancreatitis. For patients with proteinuria ≤ 19 mg/dL or creatinine levels ≤ 1 mg/dL, the use of vancomycin increased the risk of mortality during the first 28 days in the ICU. In cases of renal dysfunction or renal failure, potassium ion accumulation is likely to occur. Our study demonstrated that for patients with severe pancreatitis whose potassium levels exceed 4 mEq/L, the use of vancomycin is associated with an increased risk of death. These results suggest that controlling potassium levels is crucial for patients with severe pancreatitis who experience renal failure when using vancomycin to manage infections. This approach will allow for effective infection control while avoiding an increase in vancomycin-related mortality risk.

This study is the first to systematically analyze the impact of vancomycin on the prognosis of patients with severe pancreatitis and to identify relevant clinical indicators through interaction and mediation analyses. It provides a theoretical basis for the ICU management of patients with severe pancreatitis, particularly when using vancomycin to control specific sources of infection. This research offers corresponding monitoring indicators to assist clinicians in determining the appropriate timing for the use of vancomycin. But this study has several limitations, primarily due to its reliance on retrospective data, highlighting the need for further validation through randomized controlled trials (RCTs). Additionally, while a relationship between vancomycin dosage and the prognosis of patients with severe pancreatitis was identified, more data are required to further explore the safe and effective dosage range. This is crucial to ensure effective infection control while minimizing vancomycin-related mortality.

## Data Availability

The original contributions presented in the study are included in the article/**Supplementary Material**. Further inquiries can be directed to the corresponding authors.
